# Automatic detection of ALS from single-trial MEG signals during speech tasks: a pilot study

**DOI:** 10.3389/fpsyg.2024.1114811

**Published:** 2024-06-06

**Authors:** Debadatta Dash, Kristin Teplansky, Paul Ferrari, Abbas Babajani-Feremi, Clifford S. Calley, Daragh Heitzman, Sara G. Austin, Jun Wang

**Affiliations:** ^1^Department of Neurology, Dell Medical School, University of Texas at Austin, Austin, TX, United States; ^2^Department of Speech, Language, and Hearing Sciences, University of Texas at Austin, Austin, TX, United States; ^3^Helen DeVos Children’s Hospital, Corewell Health, Grand Rapids, MI, United States; ^4^Department of Neurology, University of Florida, Gainesville, FL, United States; ^5^MDA/ALS Center, Texas Neurology, Austin, TX, United States

**Keywords:** amyotrophic lateral sclerosis, beta oscillation, functional connectivity, magnetoencephalography, speech

## Abstract

Amyotrophic lateral sclerosis (ALS) is an idiopathic, fatal, and fast-progressive neurodegenerative disease characterized by the degeneration of motor neurons. ALS patients often experience an initial misdiagnosis or a diagnostic delay due to the current unavailability of an efficient biomarker. Since impaired speech is typical in ALS, we hypothesized that functional differences between healthy and ALS participants during speech tasks can be explained by cortical pattern changes, thereby leading to the identification of a neural biomarker for ALS. In this pilot study, we collected magnetoencephalography (MEG) recordings from three early-diagnosed patients with ALS and three healthy controls during imagined (covert) and overt speech tasks. First, we computed sensor correlations, which showed greater correlations for speakers with ALS than healthy controls. Second, we compared the power of the MEG signals in canonical bands between the two groups, which showed greater dissimilarity in the beta band for ALS participants. Third, we assessed differences in functional connectivity, which showed greater beta band connectivity for ALS than healthy controls. Finally, we performed single-trial classification, which resulted in highest performance with beta band features (∼ 98%). These findings were consistent across trials, phrases, and participants for both imagined and overt speech tasks. Our preliminary results indicate that speech-evoked beta oscillations could be a potential neural biomarker for diagnosing ALS. To our knowledge, this is the first demonstration of the detection of ALS from single-trial neural signals.

## 1 Introduction

Amyotrophic lateral sclerosis (ALS), also known as Lou Gehrig’s disease, causes rapidly progressive upper and lower motor neuron degeneration, thereby disrupting the ability of the brain to control voluntary motor function leading to dysphagia (disordered swallowing), dysarthria (disordered speech), impaired limb function, poor respiratory function, and ultimately fatality (Kiernan et al., 2011). The disease is categorized by significant across-patient heterogeneity in onset region, pattern, and rate of progression (Ravits et al., 2007). There is currently no universal standard for early detection or for monitoring the progression of ALS (Nzwalo et al., 2014; Malekzadeh, 2021). Due to the lack of a biomarker, patients with ALS are often initially misdiagnosed (up to 45% of the time) and their diagnosis can be delayed up to 12 months (Iwasaki et al., 2001).

Regardless of the focality of motor neuron degeneration at clinical onset, progressive bulbar motor deterioration is common in most patients with ALS, which leads to dysarthria (Green et al., 2013). Thus, the identification of a speech-motor biomarker for early detection of ALS has been an active area of research recently (An et al., 2018; Vieira et al., 2019; Stegmann et al., 2020). The degree to which clinicians can identify speech impairments in ALS using perceptual characteristics of speech (e.g., listening for deviations in articulation, voice quality, resonance, and prosody) is only moderately reliable (Allison et al., 2017). Early detection and monitoring of the progression of bulbar symptoms based on behavioral observations remain limited because oral-motor functional changes may not occur until muscle weakness progresses to a critical level (DePaul and Brooks, 1993; Green et al., 2013). However, physiologically, these subtle symptoms could be identified earlier by quantifying the neural activity pattern changes during speech tasks.

There have been intense investigations for diagnostic and prognostic biomarkers in the brain that can provide evidence for ALS mechanisms and thus novel targets for therapeutic intervention. Studies using functional magnetic resonance imaging (fMRI) have shown evidence of increased functional connectivity in ALS patients (Konrad et al., 2002; Lulé et al., 2007; Verstraete et al., 2010; Agosta et al., 2013). Similar findings have been reported using resting-state electroencephalography (EEG) (Iyer et al., 2015; Fraschini et al., 2016; Dukic et al., 2021) and magnetoencephalography (MEG) (Proudfoot et al., 2018; Sorrentino et al., 2018). Using MEG during a spinal motor task, another study demonstrated intensified cortical beta desynchronization followed by a delayed rebound for participants with ALS (Proudfoot et al., 2017), which hinted that beta-band oscillation may be used as an early distinguishing cortical feature for ALS. Such prior neuroimaging studies have provided tremendously impactful insights toward a better understanding of the major mechanisms of neurodegeneration due to ALS in the attempt to identify a neural biomarker. However, most neuroimaging studies have focused on group-level connectivity analyses during resting-state or spinal motor tasks. How cortical activation is impacted by ALS during speech-motor tasks has not been investigated. In addition, it is unknown if single-trial detection of ALS from neural signals is possible. In theory, single-trial ALS detection could instantly diagnose ALS in real-time thereby strengthening medical treatments for ALS. Classifying ALS on a single-trial basis involves training a machine learning model with multiple samples/trials of a quantifiable objective marker that can efficiently predict a sample/trial as ALS or healthy after proper training. Single-trial detection using machine learning has shown great potential in several neural disorders including major depressive disorder (MDD) (Liu et al., 2022), autism spectrum disorder (ASD) (Ezabadi and Moradi, 2021), post-traumatic stress disorder (PTSD) (Georgopoulos et al., 2010), schizophrenia (Xu et al., 2013), amongst other neurologic disorders (Aoe et al., 2019).

In this study, we investigated cortical differences between healthy and ALS brain signals during overt (involving bulbar motor coordination) and imagined speech (without motor involvement). The assumption is that there is a cortical disturbance during motor functions in the early stage of ALS which has been shown in previous studies (Kew et al., 1993; Mills and Nithi, 1997; Geevasinga et al., 2016; Shibuya et al., 2016; Eisen et al., 2017). Here, we used speech-motor tasks to trigger the disturbance and then detect the presence of ALS using machine learning. To our knowledge, this is the first study to use functional neuroimaging data during speech tasks for ALS detection. We examined cortical differences between healthy controls and patients with ALS using the following approaches: (1) signal correlation across sensors, (2) band power distance estimation for individual neural oscillations, (3) functional connectivity analysis, and (4) single-trial classification of ALS and healthy samples using machine learning. These approaches have been widely used in the literature to examine cortical differences between neurotypical controls and patients with neural disorders (Bob et al., 2010; Proudfoot et al., 2017, 2018; Aoe et al., 2019). Using these approaches, we found significant cortical differences between patients with ALS and healthy controls, particularly in the beta band MEG activity, which we detected at the single-trial level.

## 2 Materials and methods

### 2.1 Data collection

This study included data collected from three healthy volunteers (1 female; 52 ± 14 years) and three patients with ALS (1 female; 52 ± 12 years); see Table 1. Informed consent in accordance with the ethical committee of the participating institutions was collected from all the participants prior to data collection. The patients with ALS were in the early to mid-stage of the disease. A certified neurologist confirmed the diagnosis of ALS (one bulbar onset, one spinal onset, and one had generalized ALS symptoms). All the patients had a mild, but noticeable speech impairment (Table 1). Speech intelligibility was auditorily evaluated by a speech-language pathologist trainee who is not familiar with these patients. A commonly used software, Sentence Intelligibility Test (SIT), was used in this procedure. SIT first generated a randomized list of sentences with an increasing length from 5 to 15 words (Yorkston et al., 1996). The listener typed down what they heard from the patient’s recording in the SIT software. The software then automatically calculated the percentage of correct words (speech intelligibility) as well as speaking rate.

**Table 1:** Demographics of ALS patients.

Participant	Gender	Age (years)	Speech intelligibility (%)	Speaking rate (words/min)
A1	M	56	71.81	116.83
A2	F	39	100.00	179.45
A3	M	61	92.00	132.53

SI: Speech Intelligibility; SR: Speaking rate; wpm: words per minute.

**Figure 1:**
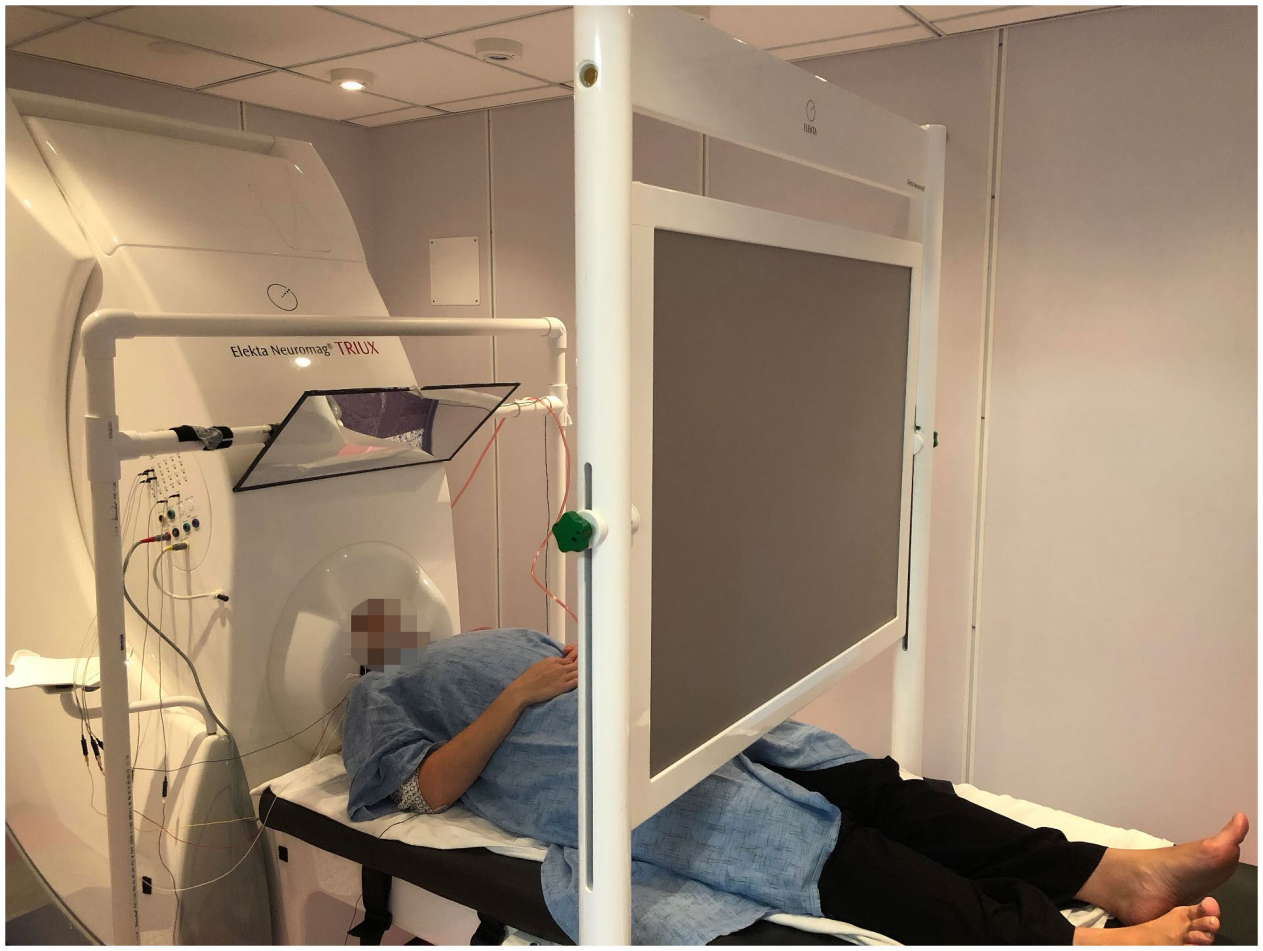
The MEG scanner and a subject with ALS.

MEG (Neuromag TRIUX; MEGIN, LCC) was used to collect the neuromagnetic signals from the participants (Figure 1). This device has 306 SQUID sensors (204 gradiometers and 102 magnetometers). A magnetically shielded room (MSIR) housed the MEG machine to restrict external magnetic noise. A digital light processing projector was used to present the visual stimuli approximately 90 cm from the subjects on a back projection screen. The stimuli were generated by a computer running the STIM2 software (Compumedics, Ltd.). Two pairs of bipolar EEG electrodes were used to record the electrocardiogram (EKG) and the electrooculogram (EOG) signals. A custom air-pressure transducer located outside the MSR and connected to the analog input of the MEG system was used to measure jaw displacement during the tasks. An air-bladder was fixed under the subjects’ chin and relayed jaw movement (via pressure on the bladder) to the transducer via tubing connected to the air-inlet on the sensor. Voice data was recorded using a standard built-in microphone connected to a transducer placed outside the MSR. Both voice and jaw movement analog signals were then digitized by feeding into the MEG ADC in real-time as separate channels. Five commonly used phrases were used as stimuli for the speech tasks: *1. Do you understand me; 2. That’s perfect; 3. How are you? Good-bye; 5. I need help.* The task phrases came from phrase lists commonly used in alternative augmented communication (AAC) devices and were selected to be more familiar to the patients and easier to recite than novel speech (Beukelman et al., 1984; Dash et al., 2020). The experiment was designed as a time-locked delayed overt reading task where each trial was time-locked to stimulus onset (display of phrases on the screen). The phrases were individually presented for 1 s in a pseudorandomized order followed by a 1 s fixation cross. The subjects were previously instructed to think of speaking the phrase without mouthing during the fixation and to overtly articulate the phrase at their normal speaking rate and loudness when the fixation disappeared. The subjects had 3 s to perform the articulation before the next stimulus trial. Each participant completed 100 trials per phrase. To overcome potential difficulties verifying the timing of imagined speech (Cooney et al., 2018), we designed our protocol to collect both speech imagination and speech production consecutively, in the same trial and under time constraints.

The MEG data were recorded with 4 kHz sampling frequency with an online filter of 0.3–1,330 Hz. The data were low pass filtered to 250 Hz with a 4^th^ order Butterworth filter and resampled to 1 kHz. Power line noise (60 Hz) and harmonics were removed with a 2^nd^ order infinite impulse response (IIR) notch filter. Only gradiometer sensors were used for analysis. From the 204 gradiometer sensors, it was observed that four sensors exhibited substantial channel noise during the data collection process from various participants. Additionally, in certain cases, one or two additional sensors displayed irregularities resembling artifacts. Consequently, a total of eight sensors were deemed unsuitable and excluded from the analysis. The discarded sensors were the same for both ALS and healthy data. Therefore, the analysis was conducted using data exclusively from 196 sensors. Independent component analysis (ICA) was used to remove artifacts (cardiac activity, eye blinks, and saccades) from the data. The continuous MEG signals were epoched into trials from −0.5 to +4.5 s centered at stimulus onset. Covert speech segment was parsed as the data from 1 s to 2 s and overt speech segment was parsed as the data from 2 s to 4.5 s of each trial. By visually inspecting the data, trials were discarded if they contained high-amplitude artifacts or if the participant did not comply with the paradigm timing (e.g., the participant spoke before being provided the cue to articulate). Jaw movement data during the covert speech segment was used to verify that the participants were not moving their articulators during the covert speech task. Jaw movement data were not used for analysis in this study. Following preprocessing, a single participant’s dataset contained only 63 valid trials for a particular phrase. Therefore, to ensure an impartial comparison, we exclusively considered the initial 60 trials per phrase per participant. The preprocessing of the raw MEG data was conducted using FieldTrip (Oostenveld et al., 2011) in MATLAB 2021b.

### 2.2 Data analysis

#### 2.2.1 Sensor correlation

Sensor correlation has been often used to characterize neurological disorders (Schindler et al., 2007; Bob et al., 2010). Here, we computed Pearson’s correlation between each pair of gradiometer sensor signals. Analyses were performed for both speech imagination and speech production for each stimulus (phrase) and participant separately. Correlation values were computed at the single-trial level and then averaged across all trials. For this analysis, we used all the spectral information (0.3–250 Hz) in the signals. Statistical 2-sample *t*-tests were used to compare the ALS and healthy groups (N = 15: 3 participants × 5 phrases) based on number of sensors showing larger absolute correlation coefficients (*r* > 0.5) and correlation density (sum of all absolute correlation values over total number of sensor-pairs) for both imagined and overt speech separately.

#### 2.2.2 Band power distance

Each neural oscillation is associated with a key functional role in the brain and could potentially carry a neural biomarker of a disorder. Beta-band power has traditionally been associated with motor function in the brain (Fisher et al., 2012; Khanna and Carmena, 2015). Thus, for the speech-motor task (overt speech) and the speech-motor imagination task (imagined speech), we compared power in this band and other canonical bands between the two groups. We computed the average power of the neuromagnetic signals for each frequency range of interest: delta (1–4 Hz), theta (4–8 Hz), alpha (8–16 Hz), beta (16–30 Hz), gamma (30–59 Hz), and high gamma (61–119 Hz). We then averaged the band powers (this was completed separately for each band) across both trials and participants. The pairwise Euclidian distances between healthy and ALS band powers were calculated across all sensors for each phrase and after averaging across the 5 phrases. 1-way analysis of variance (ANOVA) and post-hoc Tukey test was conducted with the six bands as independent groups and 5 phrases as different samples for both imagination and articulation.

#### 2.2.3 Functional connectivity

Functional connectivity is defined as the statistical dependence among measured neural signals which explains the temporal coincidence of spatially distant neurophysiological events (Friston, 1994). Functional connectivity analysis has become the conventional choice for a better understanding of the *in vivo* pathology of ALS. In this study, we used amplitude envelope correlation (AEC) (O’Neill et al., 2015) to measure the functional connectivity for each frequency band. For single-trial functional connectivity analysis, we used a 4^th^ order Butterworth bandpass filter to first bandpass the gradiometer signals from all 196 sensors at each frequency range of interest, obtained the amplitude envelopes using Hilbert transform, and then computed the pairwise linear correlation of the amplitude envelopes across all sensors for each frequency range of interest separately. Connectivity was defined as the averaged pair-wise correlation across trials. For the individual subject analysis, first, we temporally concatenated all bandpass-filtered single trials, extracted the envelope, and then computed the correlations. We performed the AEC-based functional connectivity analysis for each phrase separately during both imagined and overt speech. A 2-sample one-sided *t*-test was conducted between healthy and ALS samples (3 subjects × 5 phrases—for each group) of functional connectivity density (sum of AEC values over total number of sensor pairs) to check for the hypothesis of whether patients with ALS show greater beta band connectivity than healthy controls.

#### 2.2.4 Single-trial classification

We used power in the six canonical frequency bands of the MEG signals as features to train a linear discriminant analysis (LDA) algorithm and classified ALS and healthy data during both speech imagination and overt speech. We trained the model separately for each frequency range of interest and separately using a wide frequency range (0.3–250 Hz) which contained spectral information from all the neural oscillations. The choice of the LDA model was inspired by our previous work on speech decoding for ALS where the LDA model performed equivalently to both support vector machines and multilayer perceptron classifiers (Dash et al., 2020) at classifying 5 phrases. The *fitcdiscr* function in the Statistical and Machine Learning Toolbox of MATLAB was used for classification. The lower sample size than the feature dimension motivated for a linear type of discriminant. The linear coefficient threshold (‘Delta’) and the amount of regularization (‘Gamma’) of the model were tuned as the hyperparameters of the model, computed based on the Bayesian optimization search using a 10-fold cross-validation on the training data. All other parameters were set to the default values of the toolbox. We used a leave one-pair out cross-validation strategy where we trained the model with all trials from 2 healthy and 2 ALS participants and tested using the remaining data from 1 healthy and 1 ALS participant, irrespective of the phrase. This was repeated until each healthy-ALS pair was tested. This led to a training data size of 1,200 trials (4 participants (2 healthy +2 ALS) × 5 phrases × 60 trials) and a test data size of 600 trials (2 participants (1 healthy +1 ALS) × 5 phrases × 60 trials) for each fold. In this manner, the trained decoder was tested with completely unseen new participant data.

## 3 Results

**Figure 2:**
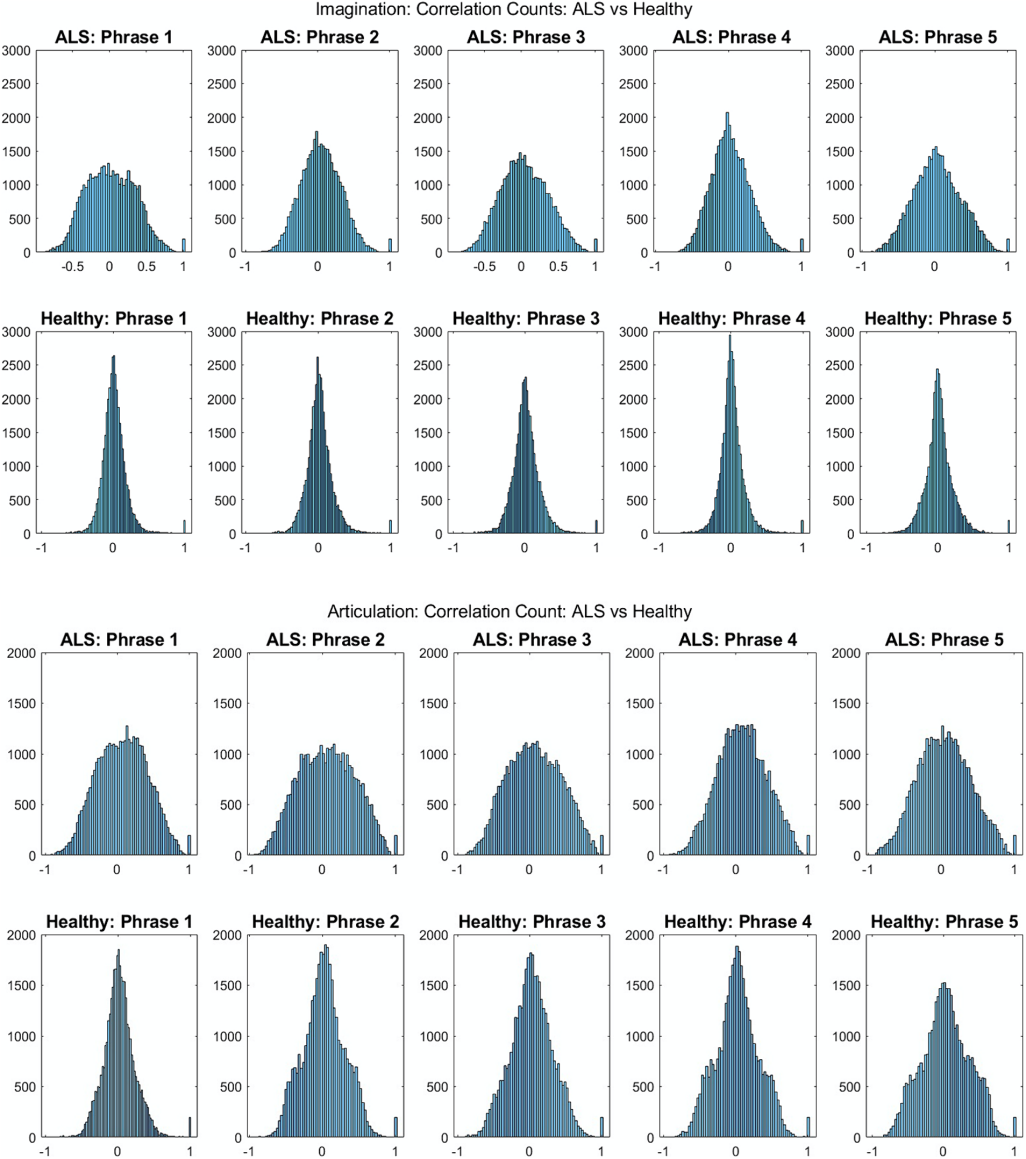
Histogram distribution of all pair-wise sensor correlations of healthy and ALS participants for each phrase during imagined speech (top) and overt speech (articulation) (bottom) for patients with ALS and healthy controls, respectively.

**Figure 3:**
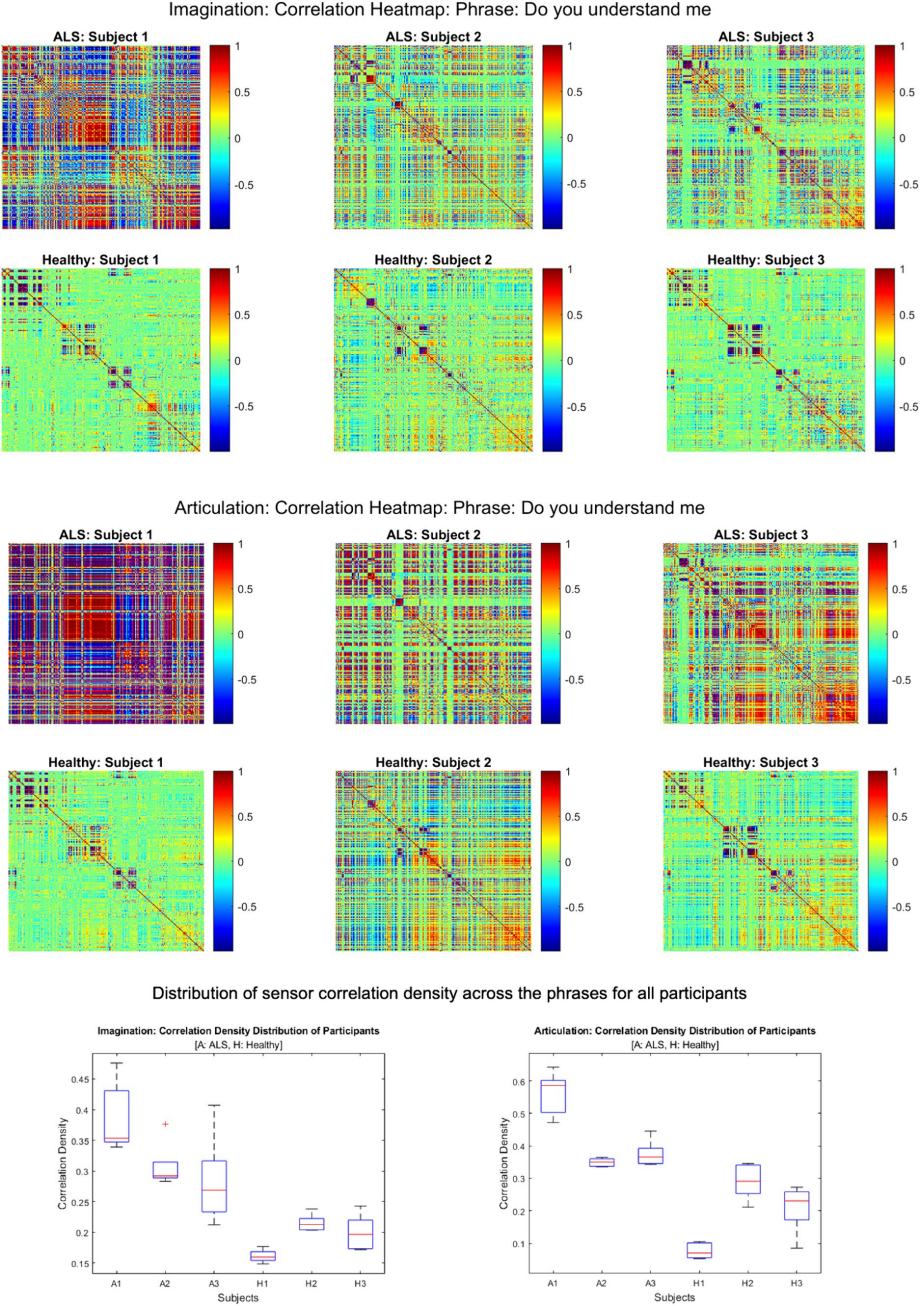
Heatmap of pair-wise sensor correlations for each subject on phrase “Do you understand me” for imagination (top) and articulation (middle), and the distribution of sensor density across the phrase for all participants (bottom). In the heatmaps, each colorful dot/point represents the correlation between a pair of the 196 gradiometers. In the bottom panel, correlation density was calculated as the sum of all absolute correlation values over total number of sensor-pairs across five phrases for each participant.

Figure 2 shows the comparative histogram distribution of sensor-level signal correlations for ALS and healthy controls for each phrase (top for imagined speech and bottom for overt speech). A significantly larger number of sensors showed greater correlations for ALS compared to healthy controls across all phrases during both overt (one-sided, 2-sample *t*-test: *t* = 3.76, *df* = 28, *p* < 0.001) and imagined speech (one-sided, 2-sample *t*-test: t = 6.01, *df* = 28, *p* < 0.001). This is also evident by the higher variance in the distribution of correlations for ALS compared to healthy controls for both imagined and overt speech across all phrases. In other words, the majority of the correlations were near mean (i.e., zero correlation) for the controls compared to ALS. For imagined speech, 95% (Bayesian analysis based on Monte Carlo simulations) of the correlation values were in a range of −0.5 to 0.5 for healthy participants. The range was between −0.8 to 0.8 for ALS participants. For overt speech, the correlation range for healthy controls was approximately within the range − 0.8 to 0.8, which was greater for the ALS ranging from −1 to 1. A heatmap plot of the correlation distribution for each subject is shown in Figure 3 (Top for imagined speech and middle for overt speech), which depicts stronger correlations across the whole brain for participants with ALS compared to the healthy controls, especially for the first participant with ALS (A1) who also had the lowest speech intelligibility and speaking rate scores (Table 1). To interpret these correlation heatmaps, correlation density was calculated as the sum of all absolute correlation values over total number of sensor-pairs and shown for each participant in Figure 3—Bottom panel. Mean correlation density was higher for participants with ALS (Overt: 0.428; Imagination: 0.326) compared to healthy subjects (Overt: 0.292; Imagination: 0.192) averaged across trials, phrases, and participants as well as statistically across all phrases and participants (one-sided, 2 sample *t*-tests: overt: *t* = 6.13, *df* = 28, *p* < 0.001; imagined: *t* = 6.68, *df* = 28, *p* < 0.001). As expected, a stronger correlation for overt speech was observed compared to speech imagination, irrespective of healthy or ALS data.

**Figure 4:**
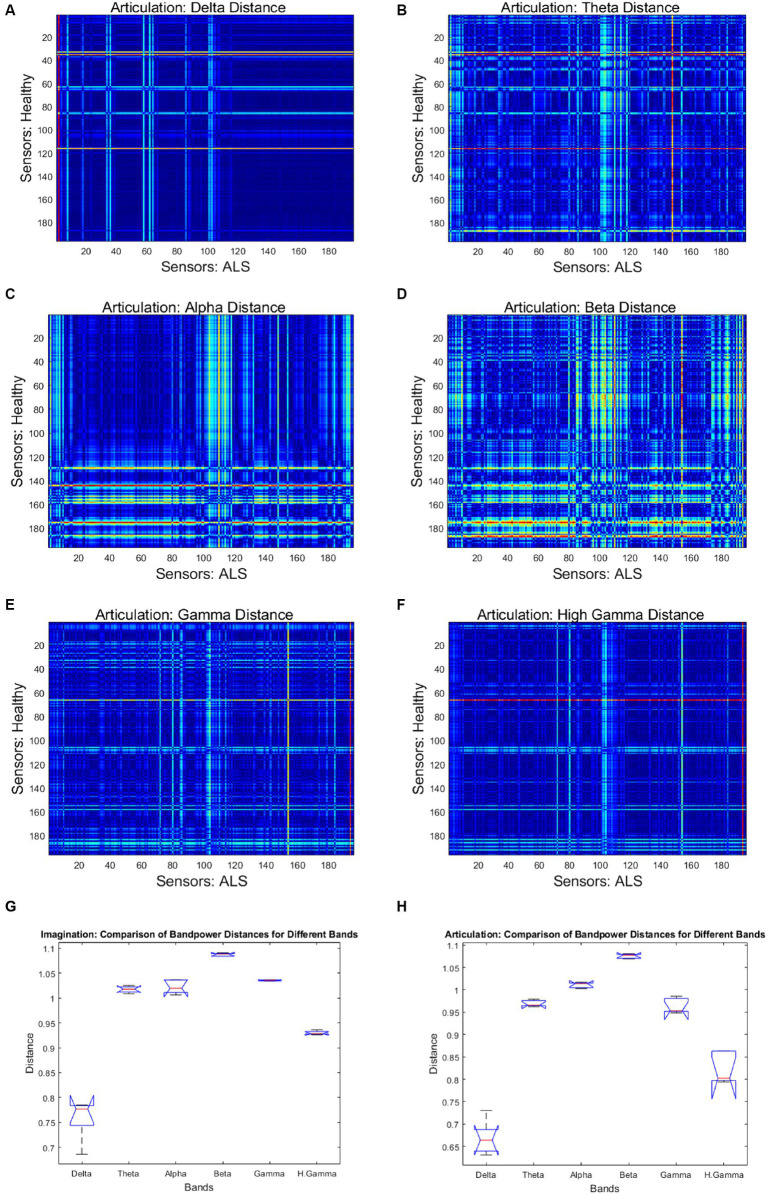
Heatmap of pairwise band-power distances between healthy and ALS sensors for each band during articulation. **(A)** Delta; **(B)** Theta; **(C)** Alpha; **(D)** Beta; **(E)** Gamma; **(F)** High Gamma. In the heatmaps, each colorful dot/point represents the bandpower distance between a pair of the 196 gradiometers. The bottom panels provide the bandpower distances for all frequency bands in speech imagination **(G)** and articulation task **(H)**, respectively.

Figure 4 shows the mean band-power distances between healthy and ALS for each during overt speech task (articulation), where panels (A) to (F) are for delta, theta, alpha, beta, gamma, and high gamma bands, respectively. For better visualization, the distances are shown as heatmaps where the color range from blue to red indicates minimum to maximum range of the normalized distance values. Each cell in the heatmap represents a pairwise distance between the band powers of a healthy sensor (y-axis) and an ALS sensor (x-axis) across all the phrases. The distances were significantly greater for the beta band powers than the other canonical bands for both imagination (1-way ANOVA: *F* = 208.55, *p* < 0.001; post-hoc Tukey tests: beta vs. rest: *p* < 0.0019) and articulation (1-way ANOVA: *F* = 206.59, *p* < 0.001; post-hoc Tukey tests: beta vs. rest: *p* < 0.0021, see Figure 4—Panel G and H). Also, a larger number of pairwise (ALS—healthy) dissimilarities were observed in the beta band. These oscillatory patterns were similar for each phrase and across all phrases for individual and group subject analysis irrespective of speech task, i.e., imagination or production. A couple of sensors showed the highest distance (solid red lines in the heatmap) which could be because those sensors were noisy.

**Figure 5:**
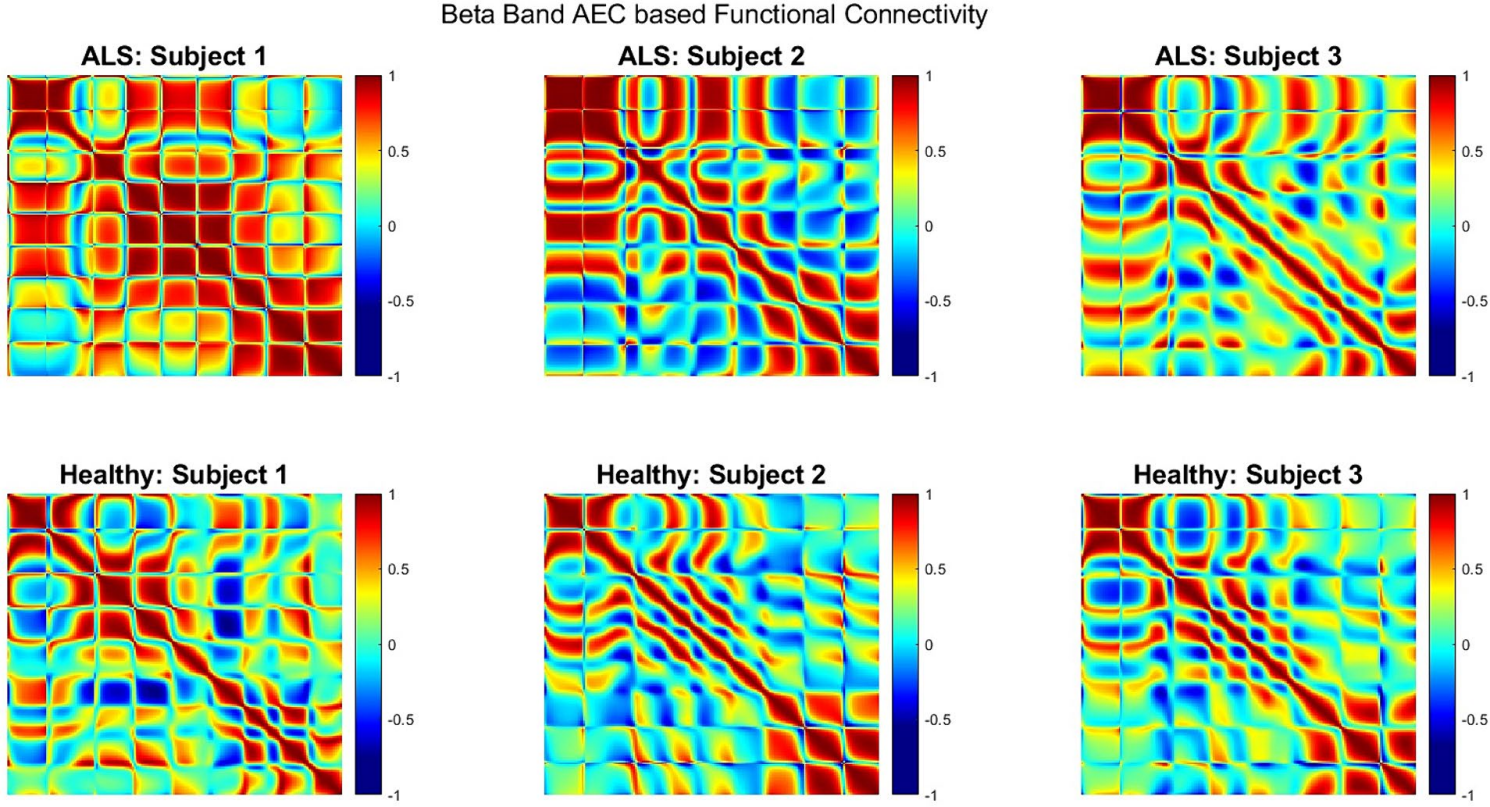
Heatmap of beta band AEC based functional connectivity across all sensors for participants with ALS (top row) and healthy controls (bottom row) during the overt speech task.

Figure 5 shows the AEC-based beta-band functional connectivity for both groups during the production of the phrase ‘*Do you understand me?*’ in the form of heatmaps; showing the correlation range of −1 to 1 (from blue [minimum] to red [maximum]). Greater beta band connectivity was significant for ALS patients compared to healthy subjects (one sided, 2-sample *t*-test: *p* < 0.05; see Supplementary Figure S3 for distributions of connectivity strengths for both patients with ALS and healthy controls). This is notably apparent in the first patient (A1), who had more severe bulbar impairment than the other two (A2 and A3). Interestingly, similar patterns of increased connectivity were also prominent during speech imagination (Supplementary Figure S2). A more diverse connectivity pattern among the 3 patients with ALS compared to the healthy participants can be observed by visualizing the connectivity strengths.

**Figure 6:**
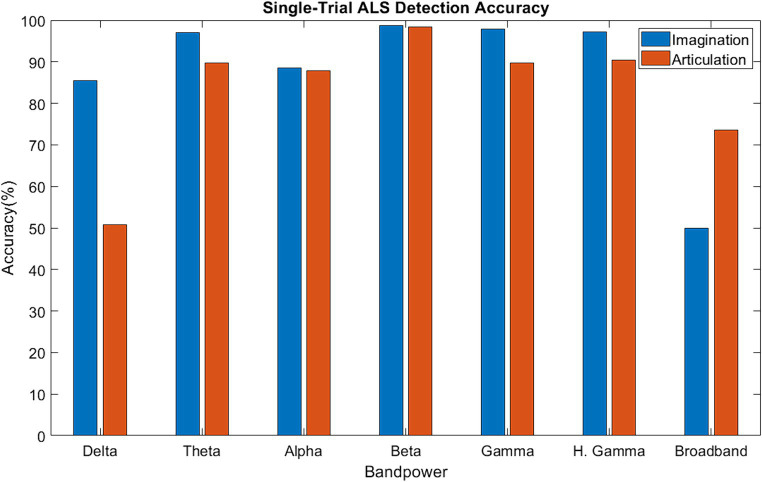
Single-trial ALS detection accuracy using band power [Delta: 1–4 Hz; Theta: 4–8 Hz; Alpha: 8–16 Hz; Beta: 16–30 Hz; Gamma: 30–59 Hz; High Gamma: 61–119 Hz; Broadband: 1–119 Hz].

Figure 6 shows the median single-trial classification accuracy for the healthy versus ALS group for both speech imagination and overt speech tasks. The best performance (median accuracy ~98%) was obtained using beta bands and was similar for both speech tasks. The performance using each individual frequency range of interest (excluding delta) was significantly higher than chance level (50%) and was also higher when compared to performance using all frequency information (all: 0.3–250 Hz). The distribution of the test performance for different folds (i.e., for each pair of ALS-healthy single-trial test accuracy) is shown in Supplementary Figure S4. The performance accuracy was lowest for the first ALS patient (A1) (mean across folds = 65% for overt speech; 83% for imagined speech), likely because this participant’s speech symptoms were severe compared to the other two participants with ALS. Although median performance was highest for beta band, statistically, 1-way ANOVA based comparison did not show a significant difference between the performances of different bands (*F* = 1.33, *p* = 0.05), possibly due to the low sample size. For the case of imagined speech, performances obtained with theta and gamma band were comparable to beta band performance.

## 4 Discussion

The evidence of greater inter-sensor correlation for ALS compared to healthy participants is a clear distinguishable marker between the two groups. This has been previously observed with M/EEG resting state (Proudfoot et al., 2019) and motor imagery studies (Yang et al., 2018). This difference in sensor correlations was apparent across all phrases and participants which further illustrates that this feature is independent of stimuli and an across-subject observation. A stronger correlation during the overt speech task compared to the imagined speech task indicated greater cortical activity for producing overt speech compared to speech imagination, which was true for both healthy and ALS groups and was expected. The signal artifacts introduced by movement during the production of speech gestures could have also contributed to the higher sensor correlation during overt speech production (Dash et al., 2018). Participant (A1) with the most severe symptoms (lowest speech intelligibility and speaking rate scores: Table 1) showed the strongest correlation (Figure 3) suggesting that the proposed approach may be useful as a marker of disease progression. We must note that a larger sample size is needed to statistically validate this observation. Further, sensor correlation differences could also arise from the differences in the head positions inside the scanner. Mapping the sensor data into source space and performing the correlations across parcels/voxels would be a better way to remove these confounds, as planned for future studies.

Beta band has been traditionally associated with motor function, and the observed differences in the beta band power during overt speech are consistent with the hypotheses that ALS is associated with cortical hyperexcitability, possibly due to the loss of inhibitory interneuron (Proudfoot et al., 2017). Our results reproduced the importance of beta band for identifying ALS during a bulbar motor task (speech). The prominent beta band differences during the speech imagination task (which does not involve motor execution) (Supplementary Figure S1) suggest that the beta band during speech tasks that involve motor planning could be a potential neural biomarker of ALS. Crucially, the pattern of band-power differences was similar for both imagination and overt speech in the beta band, possibly indicating a functional similarity between the two speech tasks. Clear differences in band power were also observed in the theta and the gamma band; however, they were less prominent compared to the beta band differences.

This finding of significantly greater beta band connectivity in the ALS group compared to healthy controls was expected since beta band functional connectivity changes have been previously shown (Verstraete et al., 2010; Agosta et al., 2013; Proudfoot et al., 2017) both during resting state as well as for spinal motor tasks. An increase in beta band functional connectivity has been hypothesized as the result from loss of intracortical inhibitory influence supported *in vivo* by neurophysiology findings of accentuated cortical beta-desynchronization during movement preparation and diminished post-movement beta-rebound (Proudfoot et al., 2017). This inhibitory influence may lead to compensatory mechanisms in early-stage ALS resulting in higher functional connectivity. Behaviorally, it may be explained as a compensatory mechanism for speech tasks in early-stage ALS due to the recruitment of larger neural networks, supported by tongue kinematic studies (Green et al., 2013; Kuruvilla-Dugdale and Mefferd, 2017; Teplansky et al., 2019). Additionally, disruption of efficient motor control networks in ALS may lead to higher cognitive control demands and attention increases, both of which are known to modulate beta-band oscillation power and connectivity (Cheyne and Ferrari, 2013; Riddle et al., 2021). This study provides the first evidence of increased beta band connectivity during a speech-motor task. Interestingly, increased connectivity was also prominent during speech imagination (Supplementary Figure S2) indicating higher beta band connectivity during speech planning, thereby strengthening the role of beta band as a neural biomarker for ALS. Similar to the observations with band-power differences, beta-band functional connectivity was greatest for our most severe patient (participant A1), providing additional confidence in the specificity of this marker. Accumulating the connectivity strength (i.e., correlation values) of all three subjects and for all 5 phrases showed greater connectivity strength for the ALS group than the control group (Supplementary Figure S2) indicating beta-band connectivity to be an across-subject marker.

Evidence of a high single-trial ALS detection accuracy with beta-band suggests that the neural mechanisms for ALS could be specific to spectral content, particularly to the beta band during speech tasks. From the previous qualitative analyses (sensor correlation, band power difference, and functional connectivity) beta-band was expected to perform the best for single-trial classification. The median accuracy with beta band was superior when both overt and imagined phrases were considered, although theta and gamma band also showed comparable performance with beta band for the case of imagined phrases. A recent study suggested covert speech emphasizes both beta and gamma band (Moon et al., 2022), which may explain why gamma band also obtained high accuracies. In short, greater performance accuracies during the speech imagination task suggest that neural signals derived while imagining speech may be optimal for diagnosing early-onset ALS, whereas overt speech may be more appropriate for evaluating the rate of disease progression. Crucially, this is the first demonstration of ALS detection from single-trial neural signals.

In terms of behavior, individuals with ALS exhibited larger onset latency and duration in overt speech tasks when compared to their healthy counterparts (2-sample t-tests, t = 3.09, *p* = 0.002, N = 900 [3 participants × 5 phrases × 60 trials]; Supplementary Figure S5), as one would expect the patients to take longer time to complete the task. It is plausible that these behavioral effects manifest in elevated sensor correlation and functional connectivity strength for ALS patients as opposed to healthy controls. However, there is notable convergence in these behaviors at the single-trial level between the two population groups, with more than 44% overlap in onset time and over 22% overlap in duration. The behavioral difference was mostly driven by the first ALS participant (A1) with the lowest speaking rate and speech intelligibility. Consequently, relying solely on behavioral indicators for single-trial detection proves to be inefficient. In addition, similar cortical differences were also observed during the covert speech task, a scenario where these behavioral markers are absent. Further, covert speech segments are immune to movement artifacts that can be present during overt speech and bias the results. Hence, the optimal approach for single-trial ALS detection involves analyzing neural activity during covert speech tasks.

Although these results are encouraging, this study suffers from a very small sample size and the omission of a non-ALS clinical control group. Future studies should include larger cohorts and include another patient population with a movement disorder, e.g., Parkinson’s disease and other motor neural diseases, in order to reveal the specificity of these detection methods. If validated, neuromagnetic signals during speech tasks with machine learning would open a new direction for assisting the diagnosis of ALS. As bulbar onset of ALS represents about 30% of the total and spinal onset accounts for about 70% (Van Es et al., 2017), we plan to combine neural signals during speech and spinal motor tasks (e.g., finger tapping) in future studies. A further step is to combine neuromagnetic signals with (speech) audio (An et al., 2018). Finally, individuals with ALS and other neurological diseases that show some similar symptoms such as Parkinson’s disease will be included for differential analysis.

## 5 Conclusion

In this study, we investigated the neuromagnetic pattern differences between individuals with ALS and healthy subjects during imagined and overt speech tasks, towards identifying a potential neural biomarker. Our preliminary results showed a greater number of sensors with larger correlations, a higher dissimilarity in the beta band power, and a larger beta band connectivity for ALS patients compared to healthy controls. Single-trial ALS detection analysis resulted in the highest median classification accuracy using beta band features, which were significant across trials, phrases, and participants for both speech imagination and articulation. The preliminary results of this study provide a proof of concept for the use of beta band as a potential neural biomarker during speech tasks and machine learning for early detection of ALS.

## Data availability statement

The raw data supporting the conclusions of this article will be made available by the authors, without undue reservation.

## Ethics statement

The studies involving humans were approved by IRBs of University of Texas at Austin and University of Texas at Dallas. The studies were conducted in accordance with the local legislation and institutional requirements. The participants provided their written informed consent to participate in this study.

## Author contributions

DD implemented the algorithms and drafted the manuscript. PF and JW designed the experimental paradigm for data collection. DH performed ALS diagnosis. DD, KT, PF, AB-F, CC, DH, SA, and JW interpreted the results and performed subsequent editing. All authors contributed to the article and approved the submitted version.

## References

[ref1] Agosta F. Canu E. Valsasina P. Riva N. Prelle A. Comi G.. (2013). Divergent brain network connectivity in amyotrophic lateral sclerosis. Neurobiol. Aging 34, 419–427. doi: 10.1016/j.neurobiolaging.2012.04.015, PMID: 22608240

[ref2] Allison K. M. Yunusova Y. Campbell T. F. Wang J. Berry J. D. Green J. R. (2017). The diagnostic utility of patient-report and speech-language pathologists’ ratings for detecting the early onset of bulbar symptoms due to ALS. Amyotroph Lateral Scler Frontotemporal Degener 18, 358–366. doi: 10.1080/21678421.2017.1303515, PMID: 28355886 PMC5530595

[ref3] An K. Kim M. Teplansky K. Green J. Campbell T. Yunusova Y.. (2018). Automatic Early Detection of Amyotrophic Lateral Sclerosis from Intelligible Speech Using Convolutional Neural Networks. Proc. Interspeech 2018, 1913–1917, doi: 10.21437/Interspeech.2018-2496

[ref4] Aoe J. Fukuma R. Yanagisawa T. Harada T. Tanaka M. Kobayashi M.. (2019). Automatic diagnosis of neurological diseases using MEG signals with a deep neural network. Sci. Rep. 9:5057. doi: 10.1038/s41598-019-41500-x, PMID: 30911028 PMC6433906

[ref9001] Beukelman D. R. Yorkston K. M. Poblete M. Naranjo C. (1984). Frequency of word occurbence in communication samples produced by adult communication aid users. J. Speech hear. disord. 49, 360–367. 6239063 10.1044/jshd.4904.360

[ref5] Bob P. Susta M. Glaslova K. Boutros N. N. (2010). Dissociative symptoms and interregional EEG cross-correlations in paranoid schizophrenia. Psychiatry Res. 177, 37–40. doi: 10.1016/j.psychres.2009.08.015, PMID: 20381169

[ref6] Cheyne D. Ferrari P. (2013). MEG studies of motor cortex gamma oscillations: evidence for a gamma “fingerprint” in the brain? Front. Hum. Neurosci. 7:575. doi: 10.3389/fnhum.2013.00575 24062675 PMC3774986

[ref7] Cooney C. Folli R. Coyle D. (2018). Neurolinguistics research advancing development of a direct-speech brain-computer interface. iScience 8, 103–125. doi: 10.1016/j.isci.2018.09.016, PMID: 30296666 PMC6174918

[ref8] Dash D. Ferrari P. Malik S. Wang J.. (2018) “Overt speech retrieval from neuromagnetic signals using wavelets and artificial neural networks,” 2018 IEEE Global Conference on Signal and Information Processing (GlobalSIP), Anaheim, CA, USA, pp. 489–493.

[ref9] Dash D. Ferrari P. Hernandez A. Heitzman D. Austin S. G. Wang J. (2020). Neural Speech Decoding for Amyotrophic Lateral Sclerosis. Proc. Interspeech 2020, 2782–2786. doi: 10.21437/Interspeech.2020-3071

[ref10] DePaul R. Brooks B. R. (1993). Multiple orofacial indices in amyotrophic lateral sclerosis. J. Speech Lang. Hear. Res. 36, 1158–1167. doi: 10.1044/jshr.3606.1158, PMID: 8114482

[ref11] Dukic S. McMackin R. Costello E. Metzger M. Buxo T. Fasano A.. (2021). Resting-state EEG reveals four subphenotypes of amyotrophic lateral sclerosis. Brain 145, 621–631. doi: 10.1093/brain/awab322, PMID: 34791079 PMC9014749

[ref12] Eisen A. Braak H. del Tredici K. Lemon R. Ludolph A. C. Kiernan M. C. (2017). Cortical influences drive amyotrophic lateral sclerosis. J Neurol Neurosurg Psychiatry 88, 917–924. doi: 10.1136/jnnp-2017-315573, PMID: 28710326

[ref13] Ezabadi M. G. Moradi M. H. (2021) ‘A novel algorithm for detection of social joint attention from single-trial EEG signals of autistic Spectrum disorder (ASD)’. In 2021 28th National and 6th International Iranian Conference on Biomedical Engineering (ICBME). IEEE, pp. 288–293.

[ref14] Fisher K. M. Zaaimi B. Williams T. L. Baker S. N. Baker M. R. (2012). Beta-band intermuscular coherence: a novel biomarker of upper motor neuron dysfunction in motor neuron disease. Brain 135, 2849–2864. doi: 10.1093/brain/aws150, PMID: 22734124 PMC3437020

[ref15] Fraschini M. Demuru M. Hillebrand A. Cuccu L. Porcu S. di Stefano F.. (2016). EEG functional network topology is associated with disability in patients with amyotrophic lateral sclerosis. Sci. Rep. 6:38653. doi: 10.1038/srep38653, PMID: 27924954 PMC5141491

[ref16] Friston K. J. (1994). Functional and effective connectivity in neuroimaging: a synthesis. Hum. Brain Mapp. 2, 56–78. doi: 10.1002/hbm.460020107

[ref17] Geevasinga N. Menon P. Özdinler P. H. Kiernan M. C. Vucic S. (2016). Pathophysiological and diagnostic implications of cortical dysfunction in ALS. Nat. Rev. Neurol. 12, 651–661. doi: 10.1038/nrneurol.2016.140, PMID: 27658852

[ref18] Georgopoulos A. P. Tan H. R. M. Lewis S. M. Leuthold A. C. Winskowski A. M. Lynch J. K.. (2010). The synchronous neural interactions test as a functional neuromarker for post-traumatic stress disorder (PTSD): a robust classification method based on the bootstrap. J. Neural Eng. 7:016011. doi: 10.1088/1741-2560/7/1/016011, PMID: 20086271

[ref19] Green J. R. Yunusova Y. Kuruvilla M. S. Wang J. Pattee G. L. Synhorst L.. (2013). Bulbar and speech motor assessment in ALS: challenges and future directions. Amyotroph Lateral Scler Frontotemporal Degener 14, 494–500. doi: 10.3109/21678421.2013.817585, PMID: 23898888 PMC3833808

[ref20] Iwasaki Y. Ikeda K. Kinoshita M. (2001). The diagnostic pathway in amyotrophic lateral sclerosis. Amyotroph Lateral Scler Other Motor Neuron Disord 2, 123–126. doi: 10.1080/146608201753275571 11771767

[ref21] Iyer P. M. Egan C. Pinto-Grau M. Burke T. Elamin M. Nasseroleslami B.. (2015). Functional connectivity changes in resting-state EEG as potential biomarker for amyotrophic lateral sclerosis. PLoS One 10:e0128682. doi: 10.1371/journal.pone.0128682, PMID: 26091258 PMC4474889

[ref22] Kew J. J. M. Leigh P. N. Playford E. D. Passingham R. E. Goldstein L. H. Frackowiak R. S. J.. (1993). Cortical function in amyotrophic lateral sclerosis: a positron emission tomography study. Brain 116, 655–680. doi: 10.1093/brain/116.3.655 8513396

[ref23] Khanna P. Carmena J. M. (2015). Neural oscillations: beta band activity across motor networks. Curr. Opin. Neurobiol. 32, 60–67. doi: 10.1016/j.conb.2014.11.010, PMID: 25528615

[ref24] Kiernan M. C. Vucic S. Cheah B. C. Turner M. R. Eisen A. Hardiman O.. (2011). Amyotrophic lateral sclerosis. Lancet (London, England) 377, 942–955. doi: 10.1016/S0140-6736(10)61156-7 21296405

[ref25] Konrad C. Henningsen H. Bremer J. Mock B. Deppe M. Buchinger C.. (2002). Pattern of cortical reorganization in amyotrophic lateral sclerosis: a functional magnetic resonance imaging study. Exp. Brain Res. 143, 51–56. doi: 10.1007/s00221-001-0981-9, PMID: 11907690

[ref26] Kuruvilla-Dugdale M. Mefferd A. (2017). Spatiotemporal movement variability in ALS: speaking rate effects on tongue, lower lip, and jaw motor control. J. Commun. Disord. 67, 22–34. doi: 10.1016/j.jcomdis.2017.05.002, PMID: 28528293 PMC5514846

[ref27] Liu M. Tan J. Jiang Y. Tian Y. (2022). Using deep learning to decode abnormal brain neural activity in MDD from single-trial EEG signals. Brain-Appar. Commun. J. Bacomics 1, 28–37. doi: 10.1080/27706710.2022.2075242

[ref28] Lulé D. Diekmann V. Kassubek J. Kurt A. Birbaumer N. Ludolph A. C.. (2007). Cortical plasticity in amyotrophic lateral sclerosis: motor imagery and function. Neurorehabil. Neural Repair 21, 518–526. doi: 10.1177/1545968307300698, PMID: 17476000

[ref29] Malekzadeh N. (2021). A comprehensive review of amyotrophic lateral sclerosis including: prevalence, pathogenesis, biomarkers diagnosis, and current treatment options. Rev. Clin. Med. 8, 180–184. doi: 10.22038/rcm.2022.57207.1365

[ref30] Mills K. R. Nithi K. A. (1997). Corticomotor threshold is reduced in early sporadic amyotrophic lateral sclerosis. Muscle Nerve 20, 1137–1141. doi: 10.1002/(SICI)1097-4598(199709)20:9<1137::AID-MUS7>3.0.CO;2-9, PMID: 9270669

[ref31] Moon J. Orlandi S. Chau T. (2022). A comparison and classification of oscillatory characteristics in speech perception and covert speech. Brain Res. 1781:147778. doi: 10.1016/j.brainres.2022.147778, PMID: 35007548

[ref32] Nzwalo H. de Abreu D. Swash M. Pinto S. de Carvalho M. (2014). Delayed diagnosis in ALS: the problem continues. J. Neurol. Sci. 343, 173–175. doi: 10.1016/j.jns.2014.06.003, PMID: 24972820

[ref33] O’Neill G. C. Barratt E. L. Hunt B. A. E. Tewarie P. K. Brookes M. J. (2015). Measuring electrophysiological connectivity by power envelope correlation: a technical review on MEG methods. Phys. Med. Biol. 60, R271–R295. doi: 10.1088/0031-9155/60/21/R271, PMID: 26447925

[ref34] Oostenveld R. Fries P. Maris E. Schoffelen J. M. (2011). FieldTrip: open source software for advanced analysis of MEG, EEG, and invasive electrophysiological data. Comput. Intell. Neurosci. 2011, 1–9. doi: 10.1155/2011/156869, PMID: 21253357 PMC3021840

[ref35] Proudfoot M. Bede P. Turner M. R. (2019). Imaging cerebral activity in amyotrophic lateral sclerosis. Front. Neurol. 9:1148. doi: 10.3389/fneur.2018.01148, PMID: 30671016 PMC6332509

[ref36] Proudfoot M. Colclough G. L. Quinn A. Wuu J. Talbot K. Benatar M.. (2018). Increased cerebral functional connectivity in ALS: a resting-state magnetoencephalography study. Neurology 90, e1418–e1424. doi: 10.1212/WNL.0000000000005333, PMID: 29661904 PMC5902786

[ref37] Proudfoot M. Rohenkohl G. Quinn A. Colclough G. L. Wuu J. Talbot K.. (2017). Altered cortical beta-band oscillations reflect motor system degeneration in amyotrophic lateral sclerosis. Hum. Brain Mapp. 38, 237–254. doi: 10.1002/hbm.23357, PMID: 27623516 PMC5215611

[ref38] Ravits J. Paul P. Jorg C. (2007). Focality of upper and lower motor neuron degeneration at the clinical onset of ALS. Neurology 68, 1571–1575. doi: 10.1212/01.wnl.0000260965.20021.47, PMID: 17485643

[ref39] Riddle J. McFerren A. Frohlich F. (2021). Causal role of cross-frequency coupling in distinct components of cognitive control. Prog. Neurobiol. 202:102033. doi: 10.1016/j.pneurobio.2021.102033, PMID: 33741402 PMC8184612

[ref40] Schindler K. Leung H. Elger C. E. Lehnertz K. (2007). Assessing seizure dynamics by analysing the correlation structure of multichannel intracranial EEG. Brain 130, 65–77. doi: 10.1093/brain/awl304, PMID: 17082199

[ref41] Shibuya K. Park S. B. Geevasinga N. Menon P. Howells J. Simon N. G.. (2016). Motor cortical function determines prognosis in sporadic ALS. Neurology 87, 513–520. doi: 10.1212/WNL.0000000000002912, PMID: 27402895

[ref42] Sorrentino P. Rucco R. Jacini F. Trojsi F. Lardone A. Baselice F.. (2018). Brain functional networks become more connected as amyotrophic lateral sclerosis progresses: a source level magnetoencephalographic study. NeuroImage: Clinical 20, 564–571. doi: 10.1016/j.nicl.2018.08.001, PMID: 30186760 PMC6120607

[ref43] Stegmann G. M. Hahn S. Liss J. Shefner J. Rutkove S. Shelton K.. (2020). Early detection and tracking of bulbar changes in ALS via frequent and remote speech analysis. NPJ Digit. Med. 3:132. doi: 10.1038/s41746-020-00335-x 33083567 PMC7555482

[ref44] Teplansky K. J. Tsang B. Y. Wang J. (2019). Tongue and lip motion patterns in voiced, whispered, and silent vowel production. In Proc. International Congress of Phonetic Sciences (pp. 1–5).

[ref45] van Es M. A. Hardiman O. Chio A. al-Chalabi A. Pasterkamp R. J. Veldink J. H.. (2017). Amyotrophic lateral sclerosis. Lancet 390, 2084–2098. doi: 10.1016/S0140-6736(17)31287-4 28552366

[ref46] Verstraete E. van den Heuvel M. P. Veldink J. H. Blanken N. Mandl R. C. Hulshoff Pol H. E.. (2010). Motor network degeneration in amyotrophic lateral sclerosis: a structural and functional connectivity study. PLoS One 5:e13664. doi: 10.1371/journal.pone.0013664, PMID: 21060689 PMC2965124

[ref47] Vieira H. Costa N. Sousa T. Reis S. Coelho L. (2019). Voice-based classification of amyotrophic lateral sclerosis: where are we and where are we going? A systematic review. Neurodegener. Dis. 19, 163–170. doi: 10.1159/000506259, PMID: 32126556

[ref48] Xu T. Stephane M. Parhi K. K. (2013) ‘Classification of single-trial MEG during sentence processing for automated schizophrenia screening’. In 2013 6th International IEEE/EMBS Conference on Neural Engineering (NER). IEEE, pp. 363–366.

[ref49] Yang T. Ang K. Phua K. S. Yu J. Toh V. Ng W.. (2018) ‘EEG channel selection based on correlation coefficient for motor imagery classification: a study on healthy subjects and ALS patient’. In 2018 40th Annual International Conference of the IEEE Engineering in Medicine and Biology Society (EMBC). IEEE, pp. 1996–1999. 10.1109/EMBC.2018.8512701 30440791

[ref9002] Yorkston K. M. Strand E. A. Kennedy M. R. (1996). Comprehensibility of dysarthric speech: Implications for assessment and treatment planning. Am J Speech Lang Pathol, 5, 55–66.

