# Supplementary material for: Automatic detection of ALS from single-trial MEG signals during speech tasks: a pilot study
Source: Front Psychol. 2024 Jun 6;15:1114811. doi: 10.3389/fpsyg.2024.1114811 (PMC11188989; doi:10.3389/fpsyg.2024.1114811)
Supplement: Supplementary file 1 [file Data_Sheet_1.docx]

Supplementary Materials


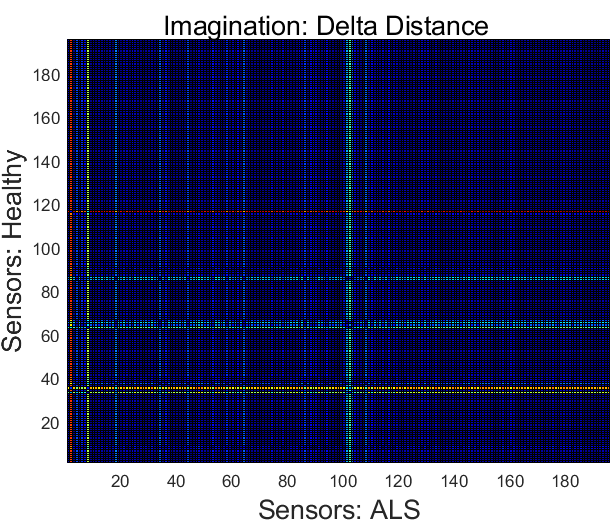

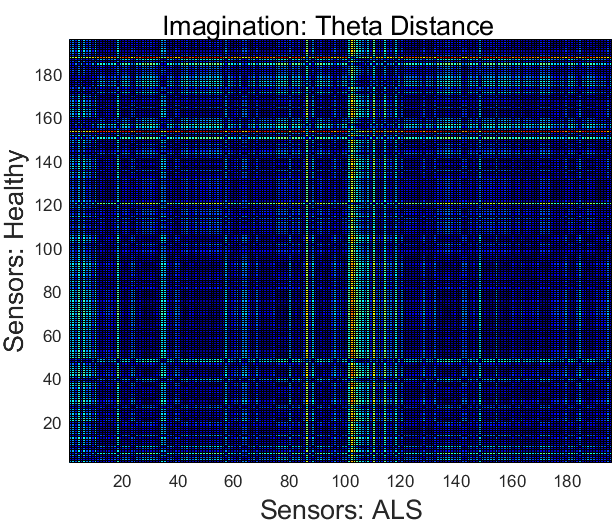

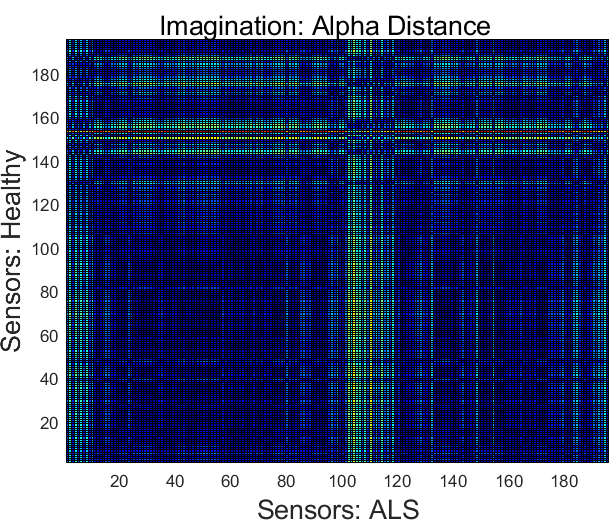

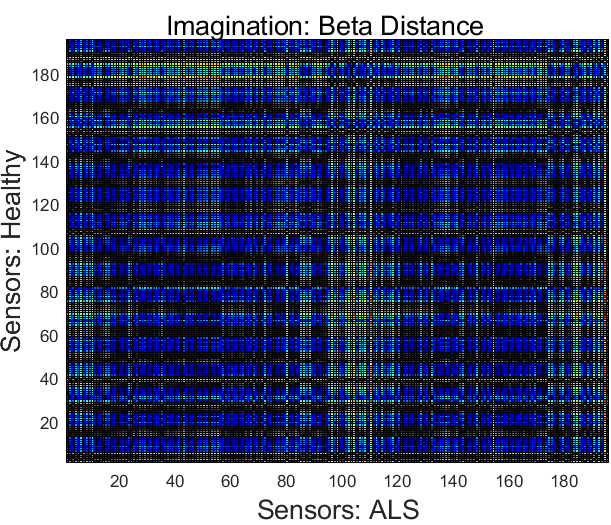

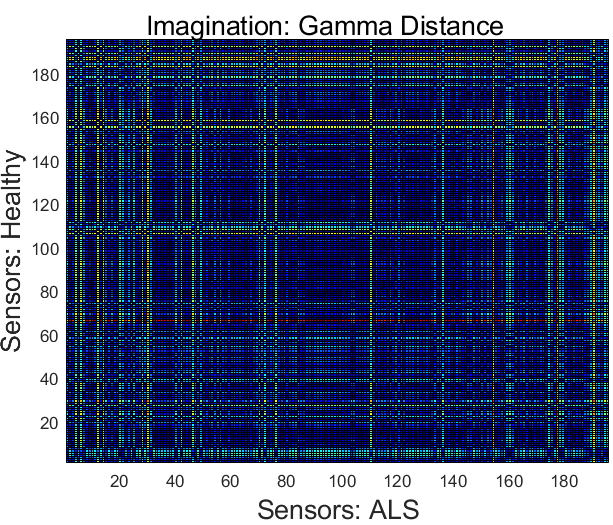

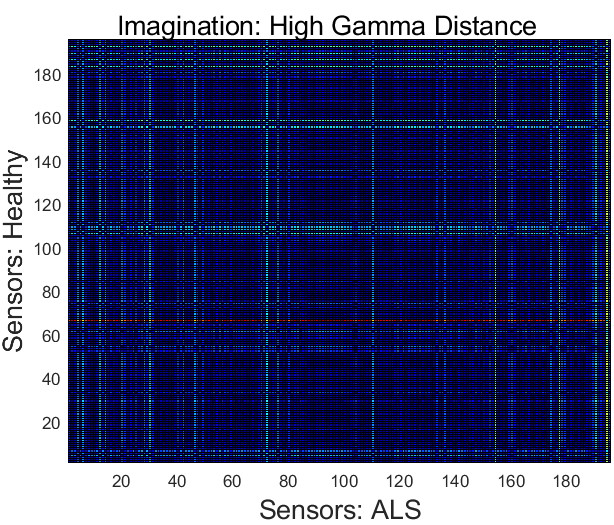


**Supplementary Figure S1** Heatmaps showing band power differences between healthy and ALS during imagined speech production.


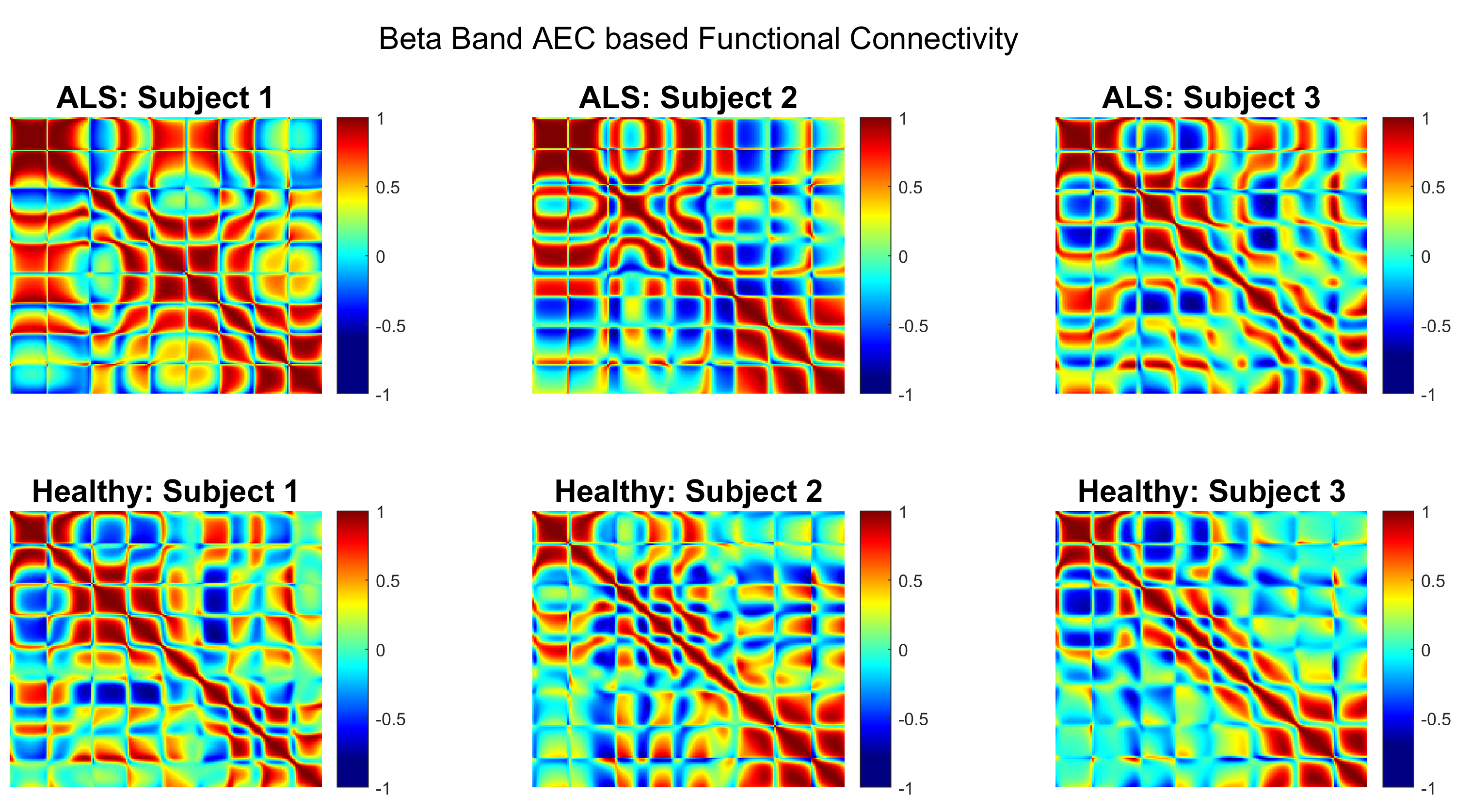


**Supplementary Figure S2.** Heatmap of beta band AEC based functional connectivity across all sensors for participants with ALS (top row) and healthy controls (bottom row) during the imagined speech task


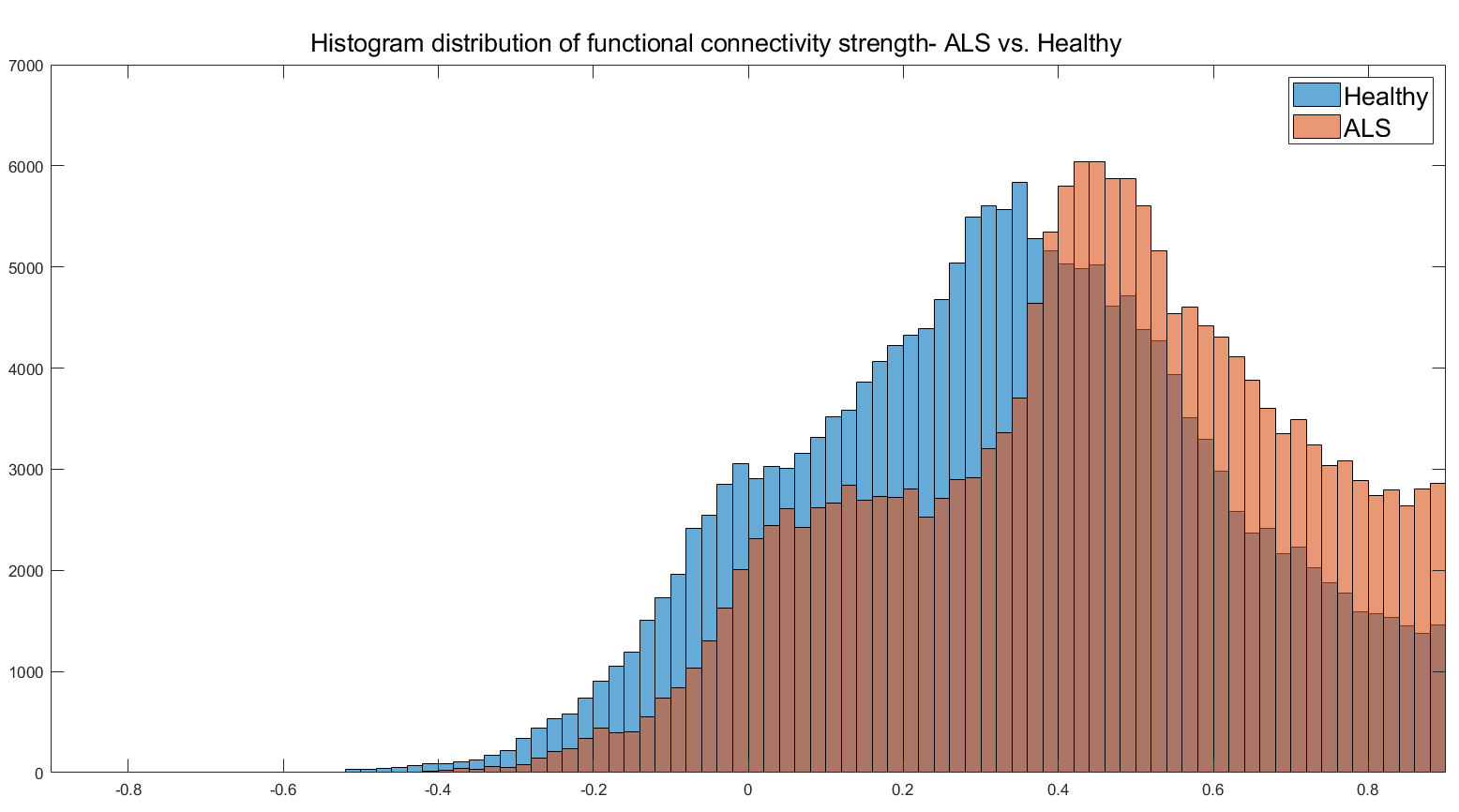


**Supplementary Figure S3.** Histogram distribution of connectivity strength for ALS and healthy group after accumulating all connectivity values of all subjects for all phrases. Y-axis shows the total sensor count of the respective correlation values (x-axis).


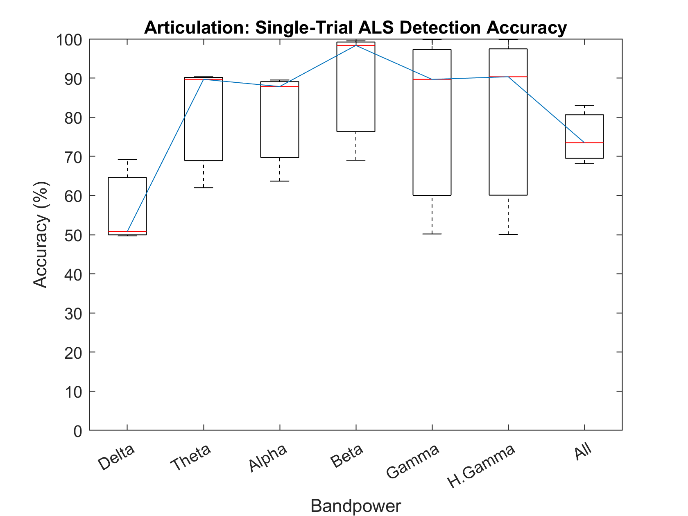

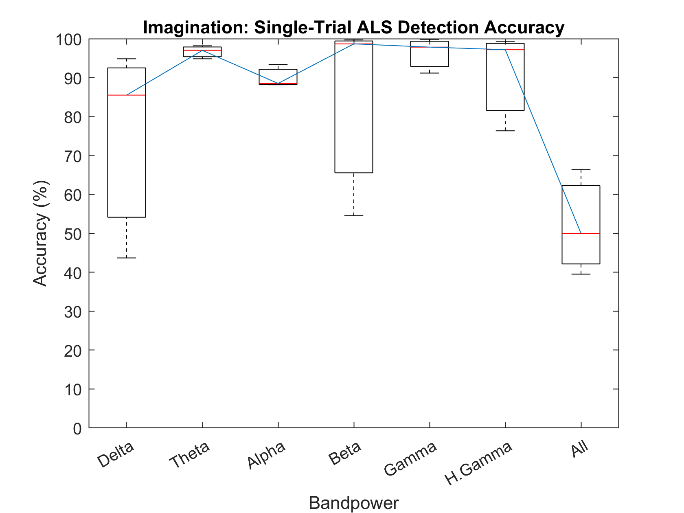


**Supplementary Figure S4.** Box plot of single-trial ALS vs. healthy classification accuracy distribution across all leave one pair out cross validation folds.


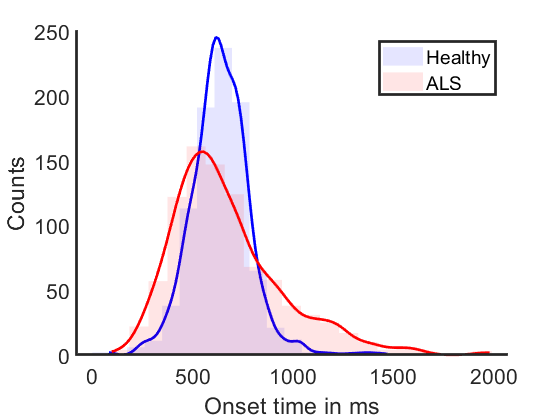


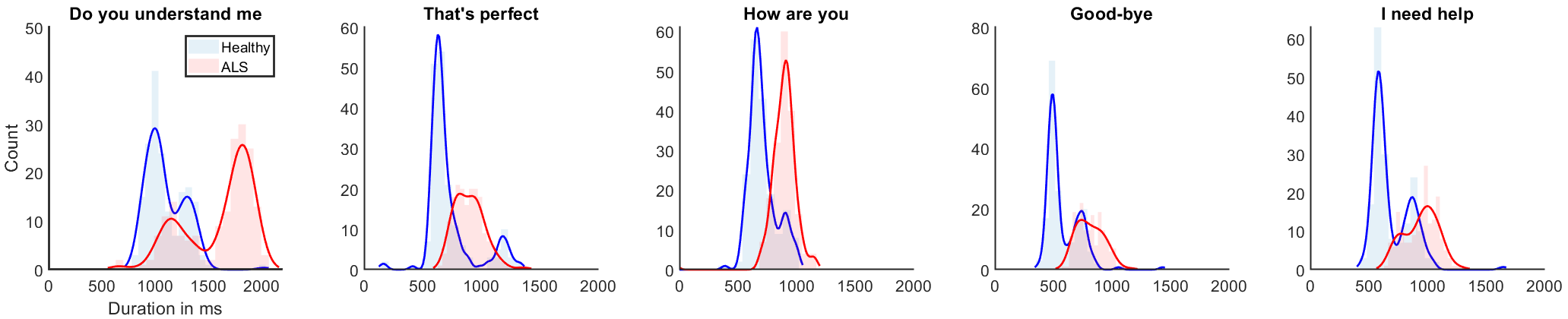


**Supplementary Figure S5. TOP.** Distribution of single-trial onset latencies for patients with ALS (red) compared to healthy controls (blue). **BOTTOM.** Distribution of single-trial speech durations for patients with ALS (red) compared to healthy controls (blue) for each phrase.
